# 

**Published:** 1893-12-30

**Authors:** 


### CONTENTS.

Ars Longa - 193
The Royal Hospital for Incurables - 194
Diseases of the Brain Due to Diseases of the Ear - 195
Famous Poisoners in Fiction—VII. Poisoner and Physician - 196
The Study of Man—III. Modern Anthropology - 197
The Field of Science - 198
Annotations.—"Ecclesiastical Evolution"—Diet for Diabetics—Complaints Against Country Hospitals - 199
Notes and News - 200
The Hospital Clinic—
Chorea - 201
London Hospital.—Treatment of Diphtheria - 202
The Infection of Phthisis - 203
New Appliances and Things Medical.—Almond Pound Cake 204
The Editor's Letter-Box.—Medical Defence Union and the London and Counties Medical Protection Society (Limited) - 204
The Institutional Workshop—
Report on Leprosy and the Trinidad Leper Asylum for 1892. By Beaven Rake, M.D., Lond. - 205
Hospitals in India—I. The Bengal Presidency - 205
Practical Departments—Window Ventilation in Sick Rooms - 206
The Story of the Insane from Year to Year - 207
Construction Notes - 207
Scraps and Gleanings - 208
The Book World of Medicine and Science - vii
Medical Vacancies and Appointments - ix

### EXTRA SUPPLEMENT.—THE NURSING MIRROR.

News from the Nursing World.—Another Plea for Our Soldiers—The Three Months' Course—A Wise Chairman—Unseemly Levity—Work for Nurses—A Valued Nurse—Amateur Classes—A Special Milk Supply—Nurses Salaries at Kimberley—Melbourne—Where are the Council?—Short Items—An Explanation Needed—Unattended Death Beds - cxxi
On the Nursing of Diseases of the Stomach.—VII. Gastric Ulcer - cxiii
An Annual Problem - cxxiii
Christmas in New York, as Spent by a Nurse - cxxiv
Christmas in India, Lucknow - cxxv
Christmas in Northern India, Amritsar - cxxv
An Unfortunate Accident—Presentation - cxxv
Christmas Entertainments - cxxvi
Our Christmas Competitions - cxxvii
For Reading to the Sick.—The Seasons—To the Old - cxxvii
Notes and Queries - cxxvii
Christmas with the Nuns. By a Worker - cxxviii
Everybody's Opinion.—Workhouse Infirmaries - cxxviii
Causerie from New England - cxxviii
St. Katharine's Hospital - cxxix
The Book World for Women and Nurses - cxxix

